# LexKO: A quick, reliable lexical test of Korean language proficiency

**DOI:** 10.3758/s13428-025-02806-z

**Published:** 2025-10-21

**Authors:** Charles B. Chang, Sunyoung Ahn, Youngjoo Kim

**Affiliations:** 1 Department of Linguistics and Translation, City University of Hong Kong, 83 Tat Chee Avenue, Kowloon, Hong Kong SAR; 2 Department of East Asian Studies, University of Toronto, 130 St. George Street, Toronto, M5S 3H1 Ontario Canada; 3 Department of Linguistics, University of Manitoba, 15 Chancellor’s Circle, Winnipeg, R3T 5V5 Manitoba Canada; 4 Department of Korean Language, Kyung Hee University, 1732 Deogyeong-daero, Yongin, Giheung-gu Gyeonggi-do Republic of Korea

**Keywords:** Korean, second language acquisition, Language learning, Multilingualism, Vocabulary, Language proficiency, Language assessment, TOPIK

## Abstract

To facilitate objective measures of proficiency for language users of diverse backgrounds, recent research in second language acquisition and multilingualism has developed short, yet reliable, tests of lexical knowledge in a wide range of languages. In this paper, we describe the development of LexKO, a brief lexically based test of Korean language proficiency, including its underlying logic, composition, intended use, and limitations. Three rounds of pilot and validation testing with first- and second-language Korean users resulted in a highly reliable Korean test comprising 60 items that can be completed in a few minutes. Freely available for other researchers to use, LexKO produces scores that correlate significantly with both first- and second-language Korean users’ scores on a standardized proficiency test (an abridged version of the Test of Proficiency in Korean) and may thus be helpful in multi-part studies for obtaining a quick, valid measure of proficiency in Korean, one of the world’s fastest-growing foreign languages.

## Introduction

Facilitated by the “Korean Wave” of cultural exports worldwide (Kuwahara, 2014; Wang and Pyun, 2020), the Korean language has seen an explosion of interest among second language (L2) learners in recent years. Despite being one of the most difficult second languages to learn for first language (L1) English speakers (Lett and O’Mara, 1990), Korean is the only language other than English for which university course enrollments in the US have consistently risen since 1980, including a fourfold increase between 2002 and 2021 (Lusin et al. , 2023, p. 44). The number of university students studying Korean as an L2 has also risen in Australia, the UK, and many other countries, reflecting a global increase in interest in the Korean language and culture (Choi, 2021; Hall and Otte, 2021; Fraschini, 2023).

Given that Korean is an increasingly common L2 worldwide, tests for reliably measuring Korean language proficiency are needed, yet there are few Korean proficiency tests that are easily accessible to the average researcher. Within the US, there used to be a proprietary SAT II Korean subject test targeted toward high school students (Seo, 2017). Currently, there are also tests targeted toward university students and professionals, such as the skill-specific Korean tests developed by the American Council on the Teaching of Foreign Languages (ACTFL) and the US government’s Interagency Language Roundtable (ILR). Within South Korea, there are two officially recognized testing options: the Korean Language Ability Test (KLAT), offered by the Korean Educational Testing Service, and the Test of Proficiency in Korean (TOPIK), offered by the National Institute for International Education. However, most of these tests take longer than one hour, and none are freely available, making them non-ideal for research pertaining to L2 users of Korean. To our knowledge, there is only one Korean test that is short enough to be useful for multi-part behavioral research on L2 users of Korean, a Korean adaptation of Lemhöfer and Broersma ’s (2012) Lexical Test for Advanced Learners of English (LexTALE). Developed by Son et al. (2022), this Korean test is also not publicly available, leaving a need for a reliable, but easily accessible, test that can provide an objective measure of Korean proficiency.

The need for objective measures of Korean proficiency dovetails with a recent move away from relying on strictly subjective proficiency measures (i.e., self-ratings of proficiency) toward including objective proficiency measures in behavioral linguistic research. The reasons for this development in the field are several, including the biasing influence of individual variables on self-ratings (see, e.g., Macintyre et al. , 1997) and disparities in rating scales; both of these factors make it difficult to interpret subjective data as directly reflecting proficiency and to compare subjective data across studies. As a result, language researchers have increasingly supplemented subjective data, collected via questionnaires such as the Language Experience and Proficiency Questionnaire (LEAP-Q; Marian et al. , 2007), with objective data, collected via brief language tasks. One such task is lexical decision, as exemplified by the above-mentioned LexTALE for English (Lemhöfer and Broersma, 2012). LexTALE is a test designed for L2 users of English, which asks the test-taker to decide whether or not each of 60 test items is a real word of English. Able to be completed in a few minutes, it provides a score ranging up to 100, which significantly correlates with test-takers’ performance in other language tasks, such as a cloze test and translation. Crucially, LexTALE was made available publicly, thus providing the research community with an accessible way of obtaining a reliable and valid measure of English proficiency; this was particularly useful to researchers conducting multi-part studies on multilingual populations, where it would not be feasible within the study design to use a lengthy proficiency test. On the model of LexTALE, Lemhöfer and Broersma (2012) created adaptations for German and Dutch. These adaptations were then followed by ones for several other languages developed by different teams of researchers: Arabic (Alzahrani, 2024), Estonian (Lõo et al., 2024), Finnish (Salmela et al., 2021), French (Brysbaert, 2013), Italian (Amenta et al., 2021), Malay (Lee et al., 2024), Mandarin Chinese (Chan and Chang, 2018; Qi et al., 2024; Wen et al., 2024), Portuguese (Zhou and Li, 2022), Sicilian (Kupisch et al., 2023), and Spanish (Izura et al., 2014).

LexTALE-type tests thus offer a way to obtain an objective, limited measure of language proficiency in empirical research on L2 learners, as opposed to relying solely on learners’ subjective impressions. The operative word here is “limited”, however, as these tests are not meant to replace longer, detailed proficiency tests in cases where a nuanced picture of proficiency is needed. Indeed, the correlation of LexTALE scores with other objective measures may vary depending on the other measures and the L1 background of the test-takers. For example, scores on the original LexTALE for English consistently showed a significant positive correlation with scores on the Quick Placement Test for English, but the correlation was considerably stronger for L1 Dutch learners ($r=0.63$) than for L1 Korean learners ($r=0.29$); additionally, LexTALE scores were less strongly correlated with scores on the Quick Placement Test than with scores on a bidirectional translation test (Lemhöfer and Broersma, 2012). This type of variation in external validity is found in other studies as well (Puig-Mayenco et al., 2023).

Crucially, such variation in validity should be expected, as LexTALE-type tests measure the breadth – and not the depth – of lexical knowledge only. In other words, when using such tests, one should note that the scores represent one aspect of lexical knowledge (namely, written word recognition), which is correlated with a learner’s holistic proficiency in a given L2 but does not represent many other components of proficiency, such as grammar and phonology. A focus on lexical breadth over depth also characterizes other lexically based tasks (e.g., translation matching or recognition), and some researchers (e.g., McLean et al. , 2020) have argued that production tasks engaging meaning recall may be better at measuring lexical knowledge than recognition tasks, such as LexTALE-type tests or the Vocabulary Size Test (Nation and Beglar, 2007). That being said, the development of LexTALE-type tests for various languages has been a significant step for L2 researchers to move beyond the traditional practice of relying solely on subjective proficiency data.

Apart from their focus on lexical breadth, another core characteristic of LexTALE-type tests, as well as other written proficiency tests, is their written nature, which has implications for their scope and interpretation. Language learning does not necessarily take place primarily through literacy in the written form of a target language, and, in fact, there may be many users of a spoken language who have substantial listening and speaking proficiency despite low reading and writing proficiency (e.g., naturalistic L2 learners, heritage speakers). Thus, some developers of LexTALE-type tests have pointed out that this type of test is meant for instructed L2 learners, excluding possibly large numbers of other users. For example, in their paper describing LEXTALE_CH for Mandarin, Chan and Chang (2018) were cautious to state explicitly that, since the test was based on Simplified Chinese characters (i.e., the writing system used in mainland China), it was not appropriate for Mandarin users who read and write primarily Traditional Chinese characters (e.g., Taiwan Mandarin users) or whose reading ability tends not to be commensurate with their overall proficiency (e.g., Chinese American heritage speakers). The use of written forms in LEXTALE_CH, however, was motivated by the relatively high rate of homophony in the Mandarin lexicon (Neergaard et al., 2022; Wen, 1980).

In the current study, we aimed to address the lack of publicly available, short proficiency tests for Korean by developing a LexTALE-type test, which we call LexKO. LexKO was intended to measure written word recognition as an index of Korean vocabulary knowledge (and, by extension, Korean proficiency) and to target L2 Korean users primarily; secondarily, we explored the application of LexKO to capturing proficiency variation among L1 Korean users. Given this focus, we presented the test items in LexKO using written forms as in other LexTALE-type tests. Although the written nature of LexKO constrains the population of Korean users it can be used with, fortunately the Korean writing system is relatively transparent (i.e., “shallow”) compared to that of languages such as Chinese and English (Marjou, 2021). Consequently, most L2 Korean learners are likely to be literate in Korean even if they never received formal instruction, meaning that LexKO may exclude fewer L2 learners based on literacy compared to other LexTALE-type tests. In the rest of this paper, we describe the development of LexKO, which consisted of two stages of pilot testing and a final stage of validation testing. We conclude the paper by comparing the performance of LexKO with that of other LexTALE-type tests and discussing best practices for proficiency measurement in empirical research on multilingualism.

## Experiment 1: Initial item assessment

### Methods

All study protocols in Experiment 1, as well as in Experiments 2 and 3, were granted exemption by the Charles River Campus Institutional Review Board of Boston University due to meeting the criteria for exemption in accordance with 45 CFR 46.104(d) 2(i)(ii) and 3(ii). The methods were performed in accordance with the ethical standards as laid down in the 1964 Declaration of Helsinki and its later amendments. Informed consent was obtained from all participants in Experiments 1–3.

#### Participants

To estimate a target sample size for Experiment 1, we conducted a power analysis for a planned one-tailed correlation of pilot test scores against proficiency ratings, using the pwrss.z.corr() function in the pwrss package (Bulus, 2023) in R (R Development Core Team, 2023). A one-tailed, as opposed to two-tailed, correlation was planned because, following the vast majority of research on language proficiency tests, our test scale was theorized to correlate with proficiency in one specific (positive) direction, and not in the opposite direction or in either of two possible directions. Taking the average correlation observed in Lemhöfer and Broersma (2012) between LexTALE scores and other proficiency measures ($r=0.5$) as our expected correlation and assuming an alpha level of 0.05 and power of 0.8, we found a target sample size of 24 participants. Therefore, we recruited L2 Korean users until we reached this sample size, along with a control group of L1 Korean users.

Participants in Experiment 1, as well as in Experiments 2–3, were recruited primarily via social media, and secondarily via in-person recruitment on a Korean university campus. Participants in Experiment 1 comprised 25 L2 Korean users (16 female, 9 male; $M_{age}=23.9$ yr, $SD=5.1$) and 50 L1 Korean users (39 female, 11 male; $M_{age}=29.1$ yr, $SD=10.6$) recruited from North America and South Korea. The L1 users were born and raised in South Korea, while the L2 users came from nine different countries spanning diverse L1 backgrounds. On a five-point Likert scale, the L2 group reported a median education level of 3 (corresponding to a bachelor’s degree), which was just above the L1 group’s median of 2 (corresponding to a high school diploma). According to mean self-ratings of Korean proficiency (averaged over separate ratings of listening, speaking, reading, and writing abilities on a five-point Likert scale), the L2 participants had intermediate to near-native Korean proficiency ($M=3.7/5$, range 2.8–5.0); this proficiency range was consistent with learners’ self-reported standardized Korean test scores, which placed them at levels 3–6 (out of six levels). The L1 participants’ self-ratings were higher and reflected having native-like Korean proficiency ($M=4.9/5$, range 4.5–5.0). The between-group difference in self-ratings was significant [Wilcoxon $W=56, p<0.001$].

#### Materials

A total of 180 items were tested in Experiment 1: 90 real and 90 nonce words. For this experiment, we started with three times as many items as in LexTALE to allow ample flexibility for final item selection while keeping the experiment a manageable length. The real words were selected to represent the diversity of the Korean lexicon and a wide range of predicted difficulty, while the nonce words were created to be phonologically and orthographically possible (i.e., to comply with all rules of Korean), to resemble the real words in length, and to vary in their phonological similarity to real words. The full list of items in Experiment 1, including metadata, is publicly available on the Open Science Framework (OSF) at https://osf.io/rf39u/.

To compile the set of real words, we started with a random selection of approximately 120 single words (twice the number of items in LexTALE) from diverse sources such as Korean books, textbooks, news websites, and other websites. We then checked their frequencies and associated proficiency levels by consulting the Modern Korean Vocabulary Frequency Dictionary (Seo, 2023) and Kim (2017); predicted word difficulty was determined based on Kim (2017) and subsequently double-checked by the second and third authors, both experienced Korean teachers. This set of words was then culled to 90, to remove overlaps in word-initials (e.g.,  ‘person’ vs.  ‘bundle’) and ensure a wide range of predicted difficulty. The culling process was carried out by the second author and her research team, and the final list was reviewed by the third author. Note that this process was not based on frequency because word frequency is not a determinative predictor of word recognition, explaining less than half of variation in word recognition performance (see, e.g., Brysbaert et al. , 2016; Brysbaert et al. , 2018). Nevertheless, in terms of number of occurrences per million *eojeol* (i.e., lexical items) in Seo (2023), the 90 real words in Experiment 1 were split across six frequency tiers: $<1.0$ ($n=30$), 1.0–5.0 ($n=22$), 5.1–10.0 ($n=14$), 10.1–20.0 ($n=14$), 20.1–100.0 ($n=8$), and >100.0 ($n=2$). They were also split across four parts of speech: noun ($n=63$), verb ($n=10$), adjective ($n=10$), and adverb ($n=7$). The length of the real words ranged from 2 to 4 syllables (median 2), and from 4 to 10 letters (median 6).

Because a large portion (57% by some estimates; Choo and O’Grady , 1996) of the Korean lexicon comprises Sino-Korean words, words of Chinese origin that have been historically integrated into Korean, a lexically based test of Korean must include Sino-Korean words to be representative of the Korean lexicon.^1^ Sino-Korean words were thus amply represented in our set of real words. Coding each real word’s Sino-Korean status, we found that 27 real words contained no Chinese base, 13 corresponded partly to a Chinese base, and 50 corresponded fully to a Chinese base. Thus, 70% of the real words were Sino-Korean. All other real words were native Korean.

The nonce words were closely matched to the real words in form. They were created broadly to resemble real words (although not necessarily by proceeding from a real word base) in order to prevent participants from using a strictly form-based (e.g., length-based) strategy to distinguish between nonce words and real words. Like the real words, the length of the nonce words ranged from 2 to 4 syllables (median 2) and from 4 to 10 letters (median 6). In addition, we coded the phonological similarity of each nonce word to real words, finding that 36 nonce words were phonological neighbors to (i.e., one phoneme change away from) a real word while 54 were not. On average, the 54 nonce words that were not phonological neighbors to a real word were 2.6 phoneme changes (*SD* 1.0; range 2–7) away from the closest real word. Therefore, the nonce words were also expected to range in difficulty.

#### Procedure

After providing informed consent, participants completed a background questionnaire and then a visual lexical decision task on the 180 items. Both the questionnaire and the lexical decision task were administered via Qualtrics (Qualtrics, 2021) on the participant’s personal device (laptop computer or smartphone). Participants were instructed to take breaks as needed. Although all tasks were self-paced, most L2 participants took less than 23 min, and most L1 participants less than 16 min, to complete the full protocol, including consent, questionnaire, and lexical decision task.

In the lexical decision task, items were presented in a checklist format, and participants were instructed to indicate, for each item, whether or not the item was a real word of Korean. In addition to saying yes for a word they knew well, they were also told to say yes “if you believe that the item is a real Korean word even if you may not know its precise meaning”. Participants were specifically instructed to respond according to their first impression and not to consult a dictionary or other tools. The items were randomized and presented on screen in Korean orthography in displays of 30 items each; thus, the task proceeded through six displays of items in all.

#### Scoring and analysis

The performance of the 180 items in Experiment 1 was assessed in two stages. First, the relationship of each item to participants’ overall responses, as indicated by their total scores out of 180, was examined using point-biserial correlation and item response theory (IRT) analysis (see, e.g., Brysbaert , 2013; Izura et al. , 2014). Second, each item was examined within the full L2 participant sample. We measured item reliability with Cronbach’s $\alpha$, and checked criterion validity by comparing the performance of the L2 and L1 groups and inspecting the one-tailed correlation of L2 participants’ performance with their self-rated proficiency.

In the first stage of analysis, we used the ltm package (Rizopoulos, 2006) in R (R Development Core Team, 2023) to assess each item’s predictiveness of overall test scores, difficulty, and discrimination power. The point-biserial correlation between participants’ responses to an item and their overall scores could range between -1 and +1, with a positive correlation indicating that a high scorer tended to perform better on the given item than a low scorer and a negative correlation indicating the opposite (i.e., an anomalous situation in which a high scorer tended to perform worse on the item than a low scorer). Items showing positive point-biserial correlations then moved on to IRT analysis, which provided information about each item’s difficulty and discrimination power (i.e., how well an item can distinguish a high scorer from a low scorer). We used the results of the IRT analysis to select a subset of items for further testing in Experiment 2.

### Results

**Fig. 1:**
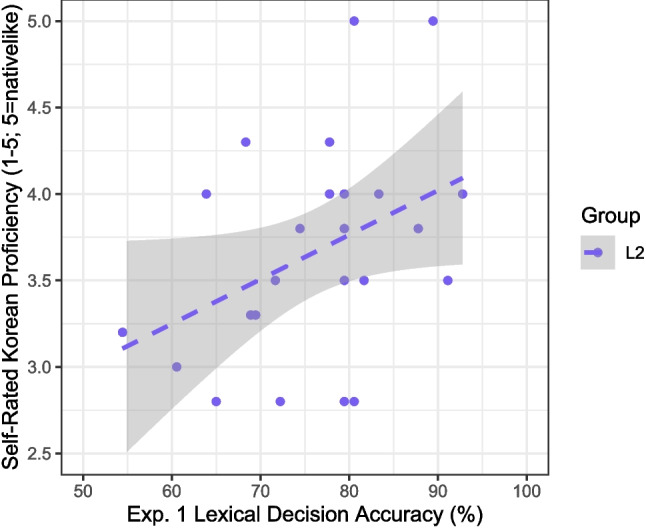
Mean self-rated Korean proficiency rating for L2 participants ($n=25$) by lexical decision accuracy in Experiment 1. The *shaded area * represents the 95% confidence interval around the regression line

All data and R analysis code from Experiment 1, as well as Experiments 2–3, are available at https://osf.io/qam4z/. L2 participants’ overall scores in the lexical decision task ranged from 98 to 167 (out of 180), corresponding to an accuracy range of 54–93% ($M=76.5\%$, $SD=9.6$). As expected, L1 participants’ overall accuracies were higher, ranging from 62% to 98% ($M=89.1\%$, $SD=9.6$). The group difference in accuracy was significant [Wilcoxon $W=199.5$, $p<0.001$]. As shown in Fig. 1, L2 participants’ accuracies were moderately correlated with their self-rated Korean proficiency [$r(22)=0.40$, 95% CI: [0.004, 0.69], $p=0.027$]. Additionally, the set of 180 items was highly reliable, over the L1 and L2 groups together and over the L2 group only [Cronbach’s $\alpha =0.97$ in both cases]. In short, the 180-item lexical decision task was both reliable and effective at distinguishing the L2 and L1 groups.

Out of the 180 items in Experiment 1, 58 showed a negative point-biserial correlation (ranging from -0.61 to -0.02), meaning that they did better with low scorers than with high scorers. These items comprised 57 real words and one nonce word. Given that these items did not predict overall scores in Experiment 1 in the desired way, we removed most of them from consideration in Experiment 2. In particular, the 45 real words with the most negative point-biserial correlations, as well as the one nonce word, were not carried forward to Experiment 2, allowing us to retain half of the Experiment 1 items (45 real words, 45 nonce words) for further testing in Experiment 2 without introducing new, untested items. We did not replace the 12 remaining real words with negative point-biserial correlations with different words to eliminate the possibility of new context (i.e., untested items that did not occur in Experiment 1) inadvertently influencing how the same items would be evaluated in Experiment 2.

Because many more nonce words showed positive point-biserial correlations than were needed in Experiment 2, we selected a subset of 45 nonce words to carry forward based on their difficulty and discrimination power as well as their performance with L1 participants. Difficulty values over all items with positive point-biserial correlations ranged from -16.2 to greater than 3.9, while discrimination power values ranged from zero to greater than 76.8. The selection of nonce words was done by ordering the items by difficulty, grouping them into nine roughly equal intervals of difficulty, and then selecting the nonce words with the best discrimination power from each interval (if available). In the few cases where a nonce word was identified unexpectedly as a real word by more than 40% of the L1 participants or was discovered to resemble phrases found on the internet, we assumed that the item was misclassified as a nonce word and dropped it from further consideration. The 45 nonce words carried forward to Experiment 2 thus represented the range in observed difficulty in Experiment 1 while showing good discrimination power and non-word identity for the vast majority of L1 participants.

To check that removing items (i.e., shortening the test) did not systematically degrade the sensitivity of the test to variation in (self-rated) Korean proficiency, we carried out a comparative analysis of the validity of L2 participants’ scores in Experiment 1, with and without the items that were ultimately removed from further testing in Experiment 2. As discussed above, accuracies on the full set of 180 test items showed a significant, positive correlation with participants’ self-rated Korean proficiency [$r=0.40$]. Crucially, accuracies on the 90 test items carried forward to Experiment 2 also showed a significant, positive correlation with self-rated Korean proficiency, which was only slightly smaller in effect size [$r(22)=0.35$ $[-0.05, 0.66]$, $p=0.045$]. In short, this analysis showed a similar ability of the smaller set of items to predict self-rated proficiency in Experiment 1, supporting the view that removing underperforming items does not generally degrade test performance.

## Experiment 2: Further item assessment

### Methods

Experiment 2 focused on narrowing down the set of 90 items carried forward from Experiment 1 to a set of approximately 60 items (i.e., a similar length of test as in the original LexTALE; Lemhöfer and Broersma , 2012). Because the overall aim of finding the strongest items for testing L2 Korean proficiency was the same as in Experiment 1, we followed the same procedure in Experiment 2.

#### Participants

Participants in Experiment 2 comprised 72 L2 Korean users (57 female, 15 male; $M_{age}=26.7$ yr, $SD=5.1$), 25 L1 Korean users (15 female, 10 male; $M_{age}=25.9$ yr, $SD=9.2$), and two female heritage language (HL) Korean users ($M_{age}=21.0$ yr, $SD=1.4$). By HL users, or “heritage speakers”, we refer to language users who grew up with early exposure to the target language as a minority language at home, within a society where a different majority language was dominant (Polinsky and Kagan, 2007; Chang, 2021). Thus, in the case of Korean, this typically refers to the Korean diaspora, such as Korean Americans or Korean Canadians. Like Experiment 1, Experiment 2 focused on true L2 users of Korean; however, rather than exclude the HL users from the discussion, we present their data separately from the L2 group, highlighting both similarities and differences between the two groups.

All participants in Experiment 2 were residing in South Korea at the time of testing. The L1 users were again born and raised in South Korea, while the L2 users came from diverse regions, including Brazil, mainland China, Hong Kong, Indonesia, Japan, Kenya, Kyrgyzstan, Mongolia, Poland, Russia, Thailand, and Vietnam. The HL participants were both from China. On a five-point Likert scale, the L2 group reported a median education level of 4 (corresponding to a master’s degree), which was just above the L1 group’s and the HL group’s median of 3.5 (between a bachelor’s degree and a master’s degree). According to mean self-ratings, the L2 participants had low intermediate to near-native Korean proficiency ($M=3.6/5$, range 2.0–5.0), as did the HL participants ($M=3.5/5$, range 2.0–5.0); this proficiency range was again consistent with these participants’ self-reported standardized Korean test scores, which placed the L2 group at levels 4–6 (out of six levels) and the HL group at levels 4–5. The L1 participants’ self-ratings were higher and reflected having native-like Korean proficiency ($M=4.9/5$, range 4.5–5.0). The difference in self-ratings between the L2 and L1 groups was significant [Wilcoxon $W=650.5, p=0.031$].

#### Materials

A total of 90 items were tested in Experiment 2: 45 real and 45 nonce words. These items were selected from the items tested in Experiment 1 (see Sections “Materials” and “Scoring and analysis”).

The full list of items in Experiment 2 is available at https://osf.io/rf39u/. As in Experiment 1, the real words in Experiment 2 were split across different parts of speech (35 nouns, five verbs, four adjectives, one adverb), and the real words and the nonce words varied in length similarly (2–4 syllables, 4–10 letters). The items were split across the different intervals of difficulty observed in Experiment 1 (see Section “Results”): the real words came from intervals 1–9, and the nonce words from intervals 4–8.

#### Procedure

The overall procedure in Experiment 2 was the same as in Experiment 1, except that Experiment 2 was conducted synchronously with an experimenter present in real time to ensure that participants completed the tasks as intended. As in Experiment 1, participants were instructed to take breaks as needed. Given that the lexical decision task in Experiment 2 contained fewer items, participants generally took less time to complete the full protocol than in Experiment 1: most L2 and HL participants took less than 17 min, and most L1 participants less than 8 min.

#### Scoring and analysis

The method of scoring and analyzing the lexical decision data was the same as in Experiment 1. The main difference vis-à-vis Experiment 1 was that there was a total of 90 items as opposed to 180 items.

### Results

L2 participants’ overall scores in the lexical decision task ranged from 31 to 87 (out of 90), corresponding to an accuracy range of 34–97% ($M=68.4\%$, $SD=11.0$). L1 participants’ overall accuracies were again higher, ranging from 57% to 100% ($M=92.6\%$, $SD=9.4$). One HL participant patterned more similarly to the L2 group, with an accuracy of 59%, while the other HL participant patterned more similarly to the L1 group, with an accuracy of 88%. Although both the L1 and L2 groups showed a wider range in accuracy in Experiment 2 as compared to Experiment 1, the group difference in accuracies was still significant [Wilcoxon $W=94.5$, $p<0.001$].

**Fig. 2:**
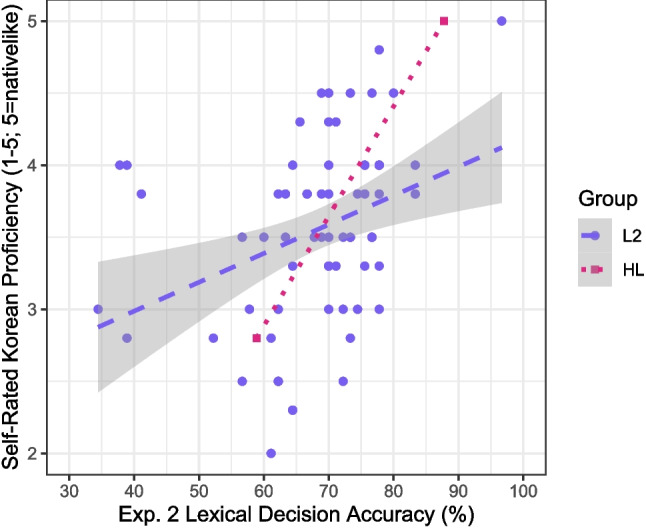
Mean self-rated Korean proficiency rating for L2 participants ($n=72$) and HL participants ($n=2$) by lexical decision accuracy in Experiment 2. The *shaded area* represents the 95% confidence interval around the regression line for the L2 group

As shown in Fig. 2, L2 participants’ accuracies again showed a significant positive correlation with their self-rated Korean proficiency, although the correlation was somewhat weaker than in Experiment 1 [$r(69)=0.36$, 95% CI: [0.13, 0.54], $p=0.001$]. Figure 2 shows further that the relationship between accuracy and self-rated proficiency for HL participants trended in a positive direction as well, although, again, there were only two HL participants. Like the set of 180 items in Experiment 1, the set of 90 items in Experiment 2 was highly reliable, over the full participant sample and over the L2 group only [Cronbach’s $\alpha =0.91$ in both cases].

Out of the 90 items in Experiment 2, 30 showed a negative point-biserial correlation (ranging from -0.74 to -0.02). These 30 items comprised 27 real words and three nonce words. Given that these items did not predict overall scores in Experiment 2 in the desired way, we removed most of them – the 25 real words with the most negative point-biserial correlations as well as all three nonce words – from further testing, leaving 20 real words and 42 nonce words.

In order to arrive at the 60-item version of LexKO that underwent validation testing in Experiment 3, we therefore removed two nonce words. As in Experiment 1, we selected the nonce words to remove and the ones to carry forward based on their difficulty and discrimination power as well as their performance with L1 participants. Difficulty values over all items with positive point-biserial correlations ranged from -3.2 to 1.4, while discrimination power values ranged from 0.2 to 4.0. As in Experiment 1, we ordered the items by difficulty, grouping them into seven intervals of difficulty, and then selected the nonce words with the weakest discrimination power in intervals 3 and 4 for removal.

Given that the original LexTALE for English contained more real words than nonce words, our decision to move forward with an item set skewed toward nonce words departed from LexTALE’s composition. We made this decision due to our finding, across Experiments 1 and 2, that L2 participants misidentified nonce words as real words at higher rates than they misidentified real words as nonce words. Because this pattern means that, provided with an even split of real and nonce words, L2 Korean users are likely to find themselves providing mostly “yes” responses, we were concerned that this bias might be noticeable and exert an undesired influence on responses (e.g., by causing participants to become stricter with “yes” responses on later items). An item set skewed toward nonce words counteracts this type of bias, so we opted to test how such an item set would perform in Experiment 3.

Following from the skew of the final item set toward nonce words, one may wonder whether this may have turned the test into merely an item discrimination task as opposed to a test of written word recognition. We do not believe so, because the test still crucially required vocabulary knowledge. First, a substantial portion of the final item set (33%) comprised real words, so obtaining a high score in Experiment 3 would require a mix of “yes” and “no” responses. Second, the real words and nonce words continued to be matched in form (see Section “Materials”), so they could not be discriminated just by looking at their phonological or orthographic composition. Therefore, in Experiment 3, we continue to interpret the test scores as reflecting visually based vocabulary knowledge and not an extraneous discrimination strategy.

## Experiment 3: Validation study

### Methods

In Experiment 3, we examined if the 60 items carried forward from Experiment 2 (i.e., LexKO) comprised a lexical test that was both reliable and valid. Testing followed the same general procedure as in Experiments 1–2. However, because one of our aims in this experiment was to check LexKO’s external validity, we included an additional task in the protocol, a standardized Korean test described further in Section “Materials”.

#### Participants

Participants in Experiment 3 comprised 53 L2 Korean users (36 female, 17 male; $M_{age}=24.4$ yr, $SD=5.7$), 58 L1 Korean users (35 female, 23 male; $M_{age}=23.7$ yr, $SD=2.9$), and one female HL Korean user (age 19 yr). As in Experiments 1–2, Experiment 3 focused on the L2 group, but we present data for the HL user alongside the L2 group’s data and discuss similarities and differences vis-à-vis the L2 group.

As in Experiment 2, all participants in Experiment 3 were residing in South Korea at the time of testing. The L1 users were born and raised in South Korea, while the L2 users again came from many different countries, including mainland China, Hong Kong, India, Indonesia, Japan, Mongolia, Myanmar, Poland, Sweden, Taiwan, Uzbekistan, Venezuela, and Vietnam. The HL participant was from China. On a five-point Likert scale, the L2 group reported a median education level of 3 (corresponding to a college degree), which matched the median in the L1 group as well as the education level of the HL participant. According to mean self-ratings, the L2 participants had low to advanced Korean proficiency ($M=2.7/5$, range 1.0–4.0); the self-ratings were consistent with self-reported standardized Korean test scores, which placed the L2 group at levels 1–6 (out of six levels). In contrast, the HL participant rated her proficiency as relatively low (1.8/5), yet had attained a recent standardized Korean test score placing her at level 6 (i.e., the top level). The L1 participants’ self-ratings were generally higher and reflected having native-like Korean proficiency ($M=4.8/5$, range 2.0–5.0). The difference in self-ratings between the L2 and L1 groups was again significant [Wilcoxon $W=113.5, p< 0.001$].

#### Materials

A total of 60 items were carried forward from Experiment 2 to comprise LexKO: 20 real and 40 nonce words. As discussed in Section “Results”, this balance of real words to nonce words is skewed toward nonce words, departing from other LexTALE-type tests, including the original LexTALE. We return to this point in Section “Scoring and analysis”.

The full list of items in LexKO is available at https://osf.io/rf39u/, and a printable version of LexKO with answer key is available at https://osf.io/45gfu/. The real words were split across different parts of speech (13 nouns, four adjectives, two verbs, and one adverb) and about evenly between native Korean words (11/20) and Sino-Korean words (9/20). The nonce words were split about evenly between being phonologically similar to a real word (18/40) and not (22/40). In terms of structure, the real and nonce words were both 2–4 syllables and 4–10 letters long, and did not differ significantly in syllable count [Wilcoxon $W=458.0, p=0.292$] or letter count [Wilcoxon $W=491.5, p=0.142$], meaning that these aspects of the items were unlikely to serve as extraneous cues in the lexical decision task.

Given our plan to check the external validity of LexKO, we added a standardized Korean test to the protocol for Experiment 3. This test was an abridged version of the Test of Proficiency in Korean (TOPIK), which we refer to as the “Mini-TOPIK”. As discussed in Section “Introduction”, the TOPIK is one of the two Korean tests officially recognized in South Korea. The Mini-TOPIK used in Experiment 3 comprised three sections corresponding to the following sections on the TOPIK: (1) vocabulary and grammar, (2) listening comprehension, and (3) reading comprehension. Each section on our Mini-TOPIK comprised ten questions; thus, the maximum score per section was ten points, and the maximum total score was 30. It was based on a similar test that has already been validated and used in research as an objective measure of Korean proficiency (Kim et al., 2012a, b, c, e, 2013, 2014; Baik and Kim, 2018), including the exact combination of 30 questions we used (Kim et al., 2012d). Our Mini-TOPIK takes less than 40 min on average to administer, so we used this test as our independent objective measure of Korean proficiency to compare to LexKO. Because it differs slightly from previous implementations in not including a writing section, we carried out post hoc analyses based on the Experiment 3 data to confirm its reliability and validity, finding that the test was indeed highly reliable [Cronbach’s $\alpha =0.93$; split-half reliability $=0.88$] and yielded scores that were significantly, and strongly, correlated with L2 participants’ overall TOPIK level [$r(32)=0.79, p<0.001$]. The questions on our Mini-TOPIK, as well as the questions on the background questionnaire in Experiment 3, can be viewed on OSF at https://osf.io/rf39u/.

#### Procedure

The procedure in Experiment 3 was the same as the procedure in Experiment 2 (i.e., consent, then questionnaire, then visual lexical decision task, with an experimenter present in real time), with two exceptions. First, LexKO presented 60 items, as opposed to 90 or 180, so the items were presented on screen at once instead of across multiple displays. This method of presentation resembled that in LEXTALE_CH (Chan and Chang, 2018). Second, as mentioned earlier, the protocol in Experiment 3 did not end with LexKO, but proceeded to an additional task, the Mini-TOPIK.

Given that the protocol in Experiment 3 contained an additional task, participants generally took more time to complete the full protocol than in Experiments 1–2. Most L2 participants took less than 53 min, while the HL participant took 46 min. As expected, the L1 participants were faster, most taking less than 24 min. That said, the LexKO component itself took only a few minutes on average.

#### Scoring and analysis

Because LexKO consists of a different number of real words and nonce words, raw accuracies do not weight performance on the two item types equally, so we followed Wen et al. (2024) in scoring LexKO by adapting the normalized Ghent score, *G*:

1 $$\begin{aligned} G = \frac{2 \times N_{yes.to.real.words} - N_{yes.to.nonce.words}}{2 \times N_{real.words}} \times 100 \end{aligned}$$

Given the 1 : 2 ratio of real words to nonce words in LexKO, the formula in Eq. 1 balances the contribution of real words (i.e., the number of “hits”, or correct “yes” responses, on real words) and the contribution of nonce words by doubling the first term ($N_{yes.to.real.words}$). Furthermore, the formula penalizes guessing by subtracting “false alarms” (i.e., the number of incorrect “yes” responses on nonce words, $N_{yes.to.nonce.words}$); the rationale for the penalty component is to produce a greater spread of scores, which can more effectively separate different proficiency levels from each other. This scoring method also follows that used in other LexTALE-type tests (e.g., Wen et al. , 2024), thereby facilitating the comparison of scores across different tests. Finally, the adjusted count of hits is divided by the adjusted total possible number of hits (i.e., twice the total number of real words; 40 for LexKO) and put on the percentage scale. Thus, as in Wen et al. (2024), LexKO scores in terms of *G* (henceforth, *G*-scores) range from -100 (for the worst case of no hits on real words and all false alarms on nonce words) to 100 (for the best case of all hits on real words and no false alarms on nonce words).

After calculating participants’ *G*-scores, we assessed the performance of LexKO in three ways. First, we measured the reliability of the test with Cronbach’s $\alpha$, and checked how the two item types were identified by the L1 group. Second, we examined criterion validity by comparing the *G*-scores of the L2 and L1 groups and inspecting the one-tailed correlation of L2 participants’ *G*-scores with their Mini-TOPIK scores. Third, we assessed the predictiveness of LexKO *G*-scores by comparing two linear regression models of Mini-TOPIK scores: one with proficiency ratings but no LexKO scores, and one with both proficiency ratings and LexKO scores as predictors. We expected that adding LexKO scores as a predictor would significantly improve upon the ratings-only model, suggesting that LexKO scores provide information about proficiency that is not captured by subjective ratings.

### Results

L2 participants’ raw scores on LexKO ranged from 20 to 45 (out of 60), corresponding to an accuracy range of 33–75% ($M=55.0\%$, $SD=9.0$). L1 participants’ accuracies were higher, ranging from 35% to 100% ($M=87.8\%$, $SD=13.2$). The HL participant patterned with the L2 group, with an accuracy of 42%. In terms of *G*-scores, the difference between the L2 group ($M=16.2$, $SD=13.7$) and the L1 group ($M=80.0$, $SD=22.3$) was even more pronounced. The group difference in *G*-scores was significant [Wilcoxon $W=119.0$, $p<0.001$]. Together, these results suggest that LexKO is not overly easy for L1 users or impossible for L2 users.

**Fig. 3:**
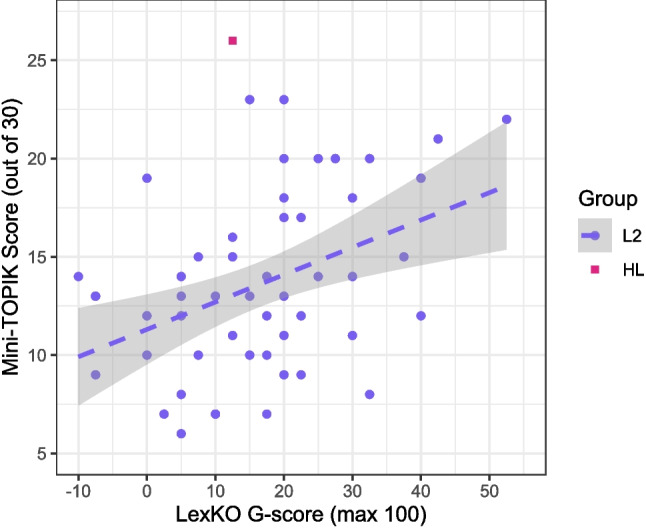
Mini-TOPIK score by LexKO score (in terms of normalized Ghent score: range -100 to 100) for L2 participants ($n=53$) and the HL participant. The *shaded area* represents the 95% confidence interval around the regression line for the L2 group

As shown in Fig. 3, L2 participants’ LexKO scores were moderately correlated with their Mini-TOPIK scores [$r(51)=0.42$ [0.17, 0.62], $p<0.001$]. Interestingly, Fig. 3 shows that the HL participant differed from most L2 participants, obtaining a high Mini-TOPIK score but a relatively low LexKO score; we return to this result in Section “Discussion”. To further probe the relationship of LexKO scores with performance on the Mini-TOPIK, we also inspected correlations with each of the three Mini-TOPIK subscores, finding that the correlation was significant for every subscore. However, the strength of the correlation varied across subscores: it was stronger with the vocabulary and grammar subscore [$r=0.44$ [0.19, 0.64]] and weaker with the listening comprehension and reading comprehension subscores [$r=0.29$ [0.02, 0.52], in both cases]. By contrast, L2 participants’ LexKO scores were only marginally correlated with their self-rated Korean proficiency [$r(51)=0.21$ $[-0.06, 0.45]$, $p=0.065$]. In short, LexKO scores show a significant positive relationship with objectively measured Korean proficiency for L2 Korean users.

The set of 60 items in LexKO showed high reliability, over the whole participant sample [Cronbach’s $\alpha =0.92$] and over the L2 group only [Cronbach’s $\alpha =0.91$]. Furthermore, all 60 items showed positive point-biserial correlations (see Section “Scoring and analysis”). With respect to how the two item types were perceived by the L1 group, all 20 of the real words were identified as such overwhelmingly (88–100% of the time), while almost all (39/40) of the nonce words were identified as such the majority of the time, suggesting that LexKO consists of a fair set of test items.

**Table 1:** Coefficients in Model 2 of Mini-TOPIK scores

Predictor	$\beta$	*SE*	95% *CI*	*t*-value	*p*-value	
(Intercept)	12.933	0.752	[11.460, 14.407]	17.202	<0.001	$$^{***}$$
Education: level 2	3.538	3.916	[-4.137, 11.213]	0.903	0.371	
Education: level 4	1.644	1.099	[-0.510, 3.798]	1.496	0.142	
Education: level 5	-0.788	2.114	[-4.933, 3.356]	-0.373	0.711	
Rating (centered)	2.384	0.693	[1.026, 3.742]	3.440	0.001	$$^{**}$$
LexKO (centered)	0.119	0.041	[0.039, 0.199]	2.906	0.006	$$^{**}$$

Significance codes: $$^{**}$$ $$p<0.01$$, $$^{***}$$ $$p<0.001$$. Model formula: MiniTOPIK $$\sim $$ Education + Rating + LexKO. The intercept represents education level 3 at the mean proficiency rating and mean LexKO *G*-score in the full participant sample

Next, we investigated the predictiveness of LexKO scores over and above self-rated Korean proficiency by comparing linear models of Mini-TOPIK scores for the L2 group, with and without their LexKO scores. Model 1, the base model, contained a treatment-coded categorical predictor for Education level (reference level = level 3, the median) and a continuous proficiency Rating predictor (centered). Model 2 contained the same predictors plus a LexKO predictor, entered as centered *G*-scores. The data met the core assumptions of linear regression (i.e., linearity, normality, non-collinearity): the dependent variable of Mini-TOPIK scores was linearly related to the focal independent variable of LexKO scores (see Fig. 3); a Shapiro-Wilk test showed that neither Mini-TOPIK scores [$W=0.957, p=0.057$] nor LexKO scores [$W=0.983, p=0.636$] deviated significantly from a normal distribution; and LexKO scores were not strongly correlated with the other predictors – only weakly with Education [Spearman’s $\rho =0.31, p=0.024$], albeit moderately with Rating [Spearman’s $\rho =0.48, p<0.001$]. The comparison of Model 2 to Model 1 revealed that adding LexKO scores as a predictor in Model 2 significantly improved upon Model 1 [$F(1,46)=8.442, p=0.006$]. Table 1 summarizes the coefficients of Model 2, which accounted for a considerable proportion of variance in Mini-TOPIK scores (adjusted $r^{2}=0.34$; cf. Model 1 adjusted $r^{2}=0.23$). Although Education was not very predictive, both Rating and LexKO significantly predicted Mini-TOPIK scores, positively in both cases. Thus, as expected, LexKO provides information about L2 Korean proficiency that is distinct from that provided by self-ratings.

Given that Lemhöfer and Broersma ’s (2012) results showed variation in LexTALE scores associated with L1 background, we also carried out an exploratory analysis of L1 effects on LexKO scores. In particular, we explored whether L2 participants from L1 backgrounds that bear some lexical connection to Korean (i.e., Chinese and Japanese, each of which contains lexical items that are primarily or secondarily, via borrowing from Chinese, related to Sino-Korean words) would show an advantage over those from L1 backgrounds that are completely unrelated. In fact, the difference in *G*-scores between these two subgroups went in the opposite direction: the 30 L1 Mandarin/Japanese L2 participants averaged 15.0 (*G*-score) on LexKO, whereas the 23 other L2 participants averaged 17.8. Crucially, this difference was not significant [Wilcoxon $W=318.5, p=0.640$], nor was the difference in their Mini-TOPIK scores [Wilcoxon $W=304.0, p=0.466$]. Although only a portion of LexKO items are Sino-Korean words, these results nevertheless suggest that the Mandarin/Japanese subgroup was not advantaged on LexKO, converging with other findings failing to show L1 effects on LexTALE-type scores (e.g., Puig-Mayenco et al. , 2023).

Because our finding of no significant difference in LexKO scores according to L1 background does not directly contradict the variation in external validity observed for LexTALE-type tests, we further explored possible variation in the validity of LexKO. In the case of the original LexTALE, correlations with scores on the general English proficiency test used (Quick Proficiency Test) varied substantially, ranging from a non-significant correlation of $r=0.12$ for intermediate learners with L1 Chinese to a significant, moderate correlation of $r=0.63$ for advanced learners with L1 Dutch (Lemhöfer and Broersma, 2012; Puig-Mayenco et al., 2023). Given the apparent relationship of this variation to L1 background and L2 proficiency, we examined the extent to which the correlation of LexKO scores with scores on the general Korean proficiency test we used (i.e., Mini-TOPIK) varied between the two L1-based subgroups mentioned above as well as between lower- and higher-proficiency subgroups. In regard to the first comparison, the correlations were both significant and the same magnitude: $r=0.42$ [0.07, 0.68], for both the L1 Mandarin/Japanese subgroup and the other L1 subgroup. In regard to the second comparison, the correlation was significant and moderate for higher-proficiency participants (i.e., those with Mini-TOPIK scores above the median; $n=23$), $r=0.40$ $[-0.01, 0.70]$, but not significant for lower-proficiency participants (i.e., those with Mini-TOPIK scores below the median; $n=23$), $r=0.23$ $[-0.19, 0.59]$. Therefore, these results converge with Puig-Mayenco et al. (2023) in suggesting that LexTALE-type tests, including LexKO, may have greater external validity for higher-proficiency than lower-proficiency L2 users.

**Fig. 4:**
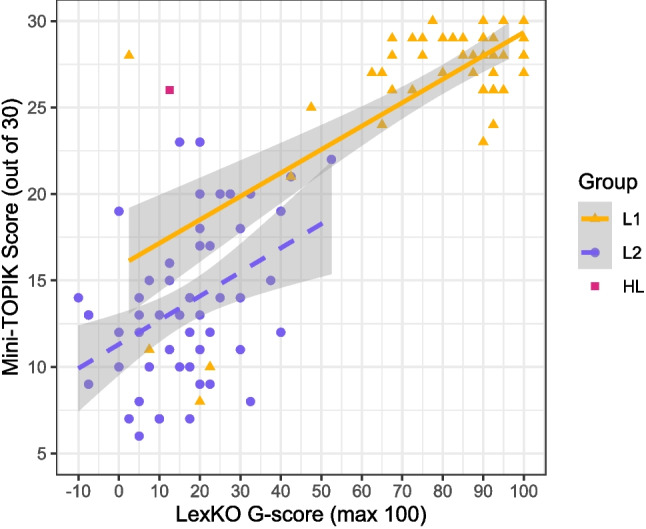
Mini-TOPIK score by LexKO score (in terms of normalized Ghent score: range -100 to 100) for all participants: L1 ($n=58$), L2 ($n=53$), and HL ($n=1$). The *shaded area* represents the 95% confidence interval around the regression line for each group

Finally, as proposed by an anonymous reviewer, we explored the potential application of LexKO as a measure of proficiency variation among L1 Korean users. For the purposes of this exploratory analysis, we first checked whether the L1 group showed substantial variation in objective Korean proficiency, as measured by Mini-TOPIK scores. Indeed, there was a considerable range in the L1 group’s Mini-TOPIK scores (8–30), as well as in their LexKO scores (2.5–100.0). Notably, L1 participants’ LexKO scores were also moderately correlated with their Mini-TOPIK scores [$r(56)=0.69$ [0.53, 0.81], $p<0.001$], as shown in Fig. 4. When four outliers (according to the 3-*SD* criterion) were excluded, the correlation was weaker, but nevertheless persisted [$r(52)=0.39$ [0.14, 0.60], $p=0.002$], and was similar in magnitude to the correlation found in the L2 group. As in the L2 group, the strength of the correlation between L1 participants’ LexKO scores and their Mini-TOPIK subscores varied across subscores: it was strongest with the vocabulary and grammar subscore [$r=0.65$ [0.47, 0.78]] and slightly weaker with the listening comprehension subscore [$r=0.64$ [0.45, 0.77]] and the reading comprehension subscore [$r=0.60$ [0.40, 0.74]], although significant ($p<0.001$) in all cases. Taken together, these results provide suggestive evidence that, insofar as a sample of L1 Korean users shows variation in Korean proficiency as in our L1 group, LexKO scores are able to reflect that variation.

## Discussion

In this study, we developed a lexically based test for obtaining a quick, yet reliable, measure of Korean proficiency – LexKO. Using the basic lexical decision paradigm as in the original LexTALE (Lemhöfer and Broersma, 2012), we considered a range of possible test items across three experiments, finding that a 60-item test comprising 20 real words and 40 nonce words yielded scores that both distinguished L2 Korean users from L1 Korean users and correlated significantly with scores on a standardized Korean test. The longer preliminary versions of our test produced scores that were already moderately correlated with self-rated proficiency; crucially, however, the final, 60-item LexKO test produced scores that were also moderately correlated with standardized Korean test scores (see Table 2). These results suggest that LexKO is a valid as well as reliable test. Given that LexKO is publicly available and takes only a few minutes to administer, it also contributes an accessible and convenient method of measuring Korean proficiency objectively.

**Table 2:** Summary of one-tailed Pearson’s correlations found for the L2 group in Experiments 1–3

Experiment	Lexical measure	Covariate	*r*	95% *CI*	df	*p*-value	
Exp. 1	percent accuracy	mean self-rating	0.40	[0.004, 0.69]	22	0.027	$^{*}$
Exp. 2	percent accuracy	mean self-rating	0.36	[0.13, 0.54]	69	0.001	$$^{**}$$
Exp. 3	LexKO *G*-score	Mini-TOPIK score	0.42	[0.17, 0.62]	51	<0.001	$$^{***}$$
Exp. 3	LexKO *G*-score	mean self-rating	0.21	$[-0.06, 0.45]$	51	0.065	

Significance codes: $$^{*}$$ $$p<0.05$$, $$^{**}$$ $$p<0.01$$, $$^{***}$$ $$p<0.001$$.

Furthermore, the performance of LexKO as a testing instrument compares favorably to that of other LexTALE-type tests. With respect to reliability, other LexTALE-type tests generally showed high values for Cronbach’s $\alpha$ ranging from 0.90 to 0.97 depending on item type and participant (sub)group (Alzahrani, 2024; Lee et al., 2024; Wen et al., 2024), and LexKO’s reliability was similarly high (Cronbach’s $\alpha =0.92$ over real and nonce words and both L1 and L2 users). With respect to validity, the LexTALE-type tests that were specifically correlated against scores on an independent general proficiency test for the target language mostly showed low to moderate correlations: $r=0.29$ to $r=0.63$ for the original LexTALE (Lemhöfer and Broersma , 2012, p. 333), $r=0.12$ to $r=0.34$ in replication testing of LexTALE (Puig-Mayenco et al. , 2023, p. 309), $r=0.39$ for LexArabic (Alzahrani , 2024, p. 5545), $r=0.29$ to $r=0.34$ for LexMAL (Lee et al. , 2024, p. 4575), and $r=0.47$ for LexCHI (Wen et al. , 2024, p. 2344). In comparison, the moderate correlations of LexKO scores with Mini-TOPIK scores ($r=0.42$ for L2 users and $r=0.69$ for L1 users) are quite strong.

Given the variation observed in the external validity of LexTALE-type tests, even for the same test (see Puig-Mayenco et al. , 2023), it is worth considering how scores on such tests should be interpreted in empirical linguistic research. Since LexTALE-type tests, including LexKO, are lexically based, it follows that scores on such tests should be most strongly correlated with measures of lexical knowledge. Indeed, we found evidence for this pattern in Experiment 3, where we observed that LexKO scores were significantly correlated with all subscores on the Mini-TOPIK, but more strongly with the vocabulary and grammar subscore than with the other two subscores related to listening and reading comprehension. This pattern reflects the fact that what LexKO measures is the presence and strength of those aspects of Korean lexical representations that support written word recognition, such as orthographic representations, and not holistic lexical quality, including many other aspects that are needed for word recognition, comprehension, and use, such as phonological, semantic, and syntactic representations (Perfetti and Hart, 2002). Given that these latter aspects are theorized to support higher-level comprehension processes, it is not surprising that LexKO scores showed weaker correlations with Mini-TOPIK reading and listening comprehension than with vocabulary and grammar. Thus, in line with Lee et al. (in press), we interpret LexKO scores as primarily reflecting knowledge of written word forms.

These observations bring us to best practices for the use of LexTALE-type tests in research on language acquisition and multilingualism. As discussed in Section “Introduction”, one of the principal contributions of LexTALE-type tests has been to provide an accessible, reliable method of objectively measuring language proficiency, as opposed to relying solely on subjective self-reports. As a reflection of L2 users’ perception, subjective self-reports of proficiency are still useful, however, and we view the role of LexTALE-type tests to be supplementing, rather than replacing, the use of subjective data. Apart from the basic goal of providing an accessible, objective measure of proficiency in a given language, another goal of the LexTALE paradigm has been to facilitate a comparative picture of proficiency for different languages, within or between participants. Here, there is potential for LexTALE-type scores to be mis- or overinterpreted, because although the various LexTALE-type tests are structured similarly, there is enough variation among them that it may be questionable to compare scores from different LexTALE-type tests to each other directly. Even when scores are put on the same scale, two LexTALE-type tests may differ in their overall difficulty, giving the same score level a different meaning across two tests. In the case of LexKO, accuracies balancing the contributions of real words and nonce words were, on average, somewhat low for LexTALE-type tests: 58% for L2 participants and 90% for L1 participants. These accuracy levels are lower than the respective accuracies of L2 participants on LexTALE (65–75%; Lemhöfer and Broersma , 2012) and LEXTALE_CH (64%; Chan and Chang , 2018) and of L1 participants on LexCHI (93%; Wen et al. , 2024), although higher than those of L2 participants on LexCHI (13%) and similar to those of L1 participants on LexTALE (89%; ibid.).

Consequently, whereas Lemhöfer and Broersma (2012) described a LexTALE balanced accuracy of 80% as a notional dividing line between “upper intermediate” (i.e., B2 on the Common European Framework of Reference) and “advanced” (C1) proficiency in L2 English, we hesitate to say that the dividing line between these proficiency levels for L2 Korean, for example, would necessarily be located at the same place on the LexKO accuracy scale. That said, after excluding one outlier, the lowest balanced accuracy on LexKO among L1 participants who scored high (i.e., at least 24/30) on the Mini-TOPIK was 81%, whereas the highest balanced accuracy among L2 participants was 76%. As shown in Fig. 3, none of the L2 participants in Experiment 3 scored above 23/30 on the Mini-TOPIK, suggesting that our L2 participant sample may have included few “advanced” learners and thus evinced lower proficiency on average than the L2 participants in Lemhöfer and Broersma (2012). If this is true, then it may be reasonable to posit 80% balanced accuracy (corresponding to a *G*-score of approximately 60) on LexKO as a dividing line between upper-intermediate and advanced proficiency in Korean. However, we are cautious to point out that this represents, for our sample, a dividing line between L1 and L2 users, and not between high-intermediate and advanced L2 users per se. Thus, further research is needed to verify how LexKO scores align with different proficiency levels within a full range of L2 Korean proficiency. As for the dividing line between “upper intermediate” (B2) and “lower intermediate” (B1), based on Fig. 3 we tentatively suggest a *G*-score of 20, meaning that participants who obtain *G*-scores below 20 will mostly be elementary or low-intermediate learners.

Returning to best practices, we recommend, if it is necessary to compare scores on different LexTALE-type tests, that researchers carefully consider the language-specific and test-specific aspects of the additional languages they are comparing, especially in relation to their learner population. It is well-known that languages differ in difficulty of acquisition for L2 learners from the same L1 background, reflected in the disparate amounts of time needed to learn different target languages to the same level (Lett and O’Mara, 1990; see Bowles et al., 2016 for further discussion). Moreover, vocabulary may be acquired at different rates in L1 development depending on the language, possibly due to linguistic characteristics such as phonology (Bleses et al., 2008). As a result, LexTALE-type tests for different languages are likely to differ in difficulty, because lexical development may not proceed at the same pace across various target languages and each LexTALE-type test is typically developed on its own without being normed in difficulty to the original LexTALE. Additionally, due to the vagaries of how different test items may perform during test development, any given LexTALE-type test may differ to some degree from the original LexTALE structurally, such as in the number of items (e.g., 90 in LextPT and LEXTALE_CH, instead of 60; Zhou and Li , 2022, Chan and Chang , 2018) or the ratio of real word to nonce word items (e.g., 1 : 2 in LexKO, instead of 2 : 1 or 1 : 1). Although this variation is not necessarily a problem for an individual LexTALE-type test, it is a problem for the direct comparison of different LexTALE-type tests to each other. Therefore, rather than assume that different LexTALE-type tests will straightforwardly line up score-wise, it is advisable to consider how an individual LexTALE-type test yields scores corresponding to specific proficiency levels in its own right before comparing scores on that test to those on another test for a different language, in the manner we have started to do above for LexKO in relation to LexTALE.

We close with remarks on limitations and future research. First, as discussed in Section “Introduction”, LexKO, like all LexTALE-type tests, is primarily a test of lexical knowledge – in particular, knowledge of word forms (see Lee et al. , in press, for further discussion). Although lexical knowledge is part of language proficiency and LexKO scores significantly correlate with general proficiency test scores, LexKO is not meant to replace detailed proficiency tests that yield nuanced measures of proficiency. The contribution of LexKO is instead to provide an accessible, reliable method of obtaining a quick objective measure of Korean proficiency, which can be used to supplement other metrics of proficiency such as self-ratings. Second, like other LexTALE-type tests, LexKO requires literacy in the target language, meaning that it is primarily a test for educated L2 Korean learners and may not be appropriate for naturalistic L2 learners or HL users (i.e., heritage speakers). However, given that HL users comprise a significant portion of non-Korean-dominant users of Korean, there is clearly a need for a quick Korean test that is better adapted for HL users’ unique linguistic profile. For example, HL users often show a “yes” bias in responses on their HL (Polinsky, 2018), which may be related to linguistic insecurity concerning their HL abilities (Driver, in press). In Experiment 3, we saw suggestive evidence of both of these characteristics in the lone HL participant, who showed objectively high Korean proficiency (as reflected in a high Mini-TOPIK score and a high self-reported external Korean test score) yet rated her Korean proficiency as low and obtained a low LexKO score (mostly due to incorrect “yes” responses on nonce words, i.e., false alarms). The HL participant thus patterned differently from others in Experiment 3 (see Fig. 3), consistent with the view that LexKO may not effectively measure proficiency for HL users. Looking forward, a challenge for developing a quick Korean test for HL users will therefore be to determine how to overcome the “yes” bias that leads to false alarms in the lexical decision paradigm or whether to adopt a different testing paradigm altogether, such as elicited imitation (see, e.g., Bowles , 2021). Future research focusing on the assessment of proficiency in HL users will contribute to enhancing the inclusivity of research on Korean, as well as research on other languages with sizable diasporic speech communities.

## Open Practices Statement

The materials for administering LexKO are available at https://osf.io/45gfu/, with the full item list including metadata at https://osf.io/rf39u/. The data and analysis code for all analyses reported in this paper are available at https://osf.io/qam4z/. None of the reported studies was preregistered.

## Data Availability

The data underlying all analyses reported in this paper are available at https://osf.io/qam4z/. The materials for administering LexKO are available at https://osf.io/45gfu/. The code for all analyses reported in this paper is available at https://osf.io/qam4z/.

## Footnotes

1 Following from their Chinese provenance, Sino-Korean words overlap in phonology and meaning with modern Chinese words. However, given that Korean and Chinese have both continued to develop and change since the time of these borrowings, there is generally some degree of phonological and/or semantic disparity between the Sino-Korean words and the corresponding words in modern varieties of Chinese, such as Mandarin (e.g., the Sino-Korean word  ‘Bible’ vs. the corresponding Mandarin Chinese word  ‘Bible’).

## Code Availability

The code for all analyses reported in this paper is available at https://osf.io/qam4z/.
